# “It’s the Seeing and Feeling”: How Embodied and Conceptual Knowledges Relate in Pipeline Engineering Work

**DOI:** 10.1007/s11133-022-09520-8

**Published:** 2022-10-20

**Authors:** Sarah Maslen, Jan Hayes

**Affiliations:** 1 Canberra School of Politics, Economics & Society, Faculty of Business, Government & Law, University of Canberra, 2617 Canberra, ACT Australia; 2 School of Property, Construction and Project Management, RMIT University, 3001 Melbourne, VIC Australia

**Keywords:** Embodiment, Engineering practice, Fieldwork, Reasoning, Risk, Senses

## Abstract

This paper examines the relationship between conceptual and embodied reasoning in engineering work. In the last decade across multiple research projects on pipeline engineering, we have observed only a few times when engineers have expressed embodied or sensory aspects of their practice, as if the activity itself is disembodied. Yet, they also often speak about the importance of field experience. In this paper, we look at engineers’ accounts of the value of field experience showing how it works on their sense of what the technology that they are designing looks, feels, and sounds like in practice, and so what this means for construction and operation, and the management of risk. We show how office-based pipeline engineering work is an exercise in embodied imagination that humanizes the socio-technical system as it manifests in the technical artifacts that they work with. Engineers take the role of the other to reason through the practicability of their designs and risk acceptability.

## Introduction

Some professionals work in a context where bad decisions can have catastrophic consequences. Major disasters such as Deepwater Horizon (National Commission on the BP Deepwater Horizon Oil Spill and Offshore Drilling 2011), Fukushima (IAEA Director General 2015), and the Boeing 737 MAX crashes (Majority Staff of the Committee on Transportation and Infrastructure 2020) have causal factors that include poor judgement on the part of key decision makers. This is not to apportion blame but rather to emphasize the importance of understanding what contributes to the best decision-making in the context of uncertainty in complex socio-technical systems. This is a matter that occupies scholars from a wide range of disciplines including engineering (Suhr 1999; Trevelyan 2014), psychology (Janis 1982; Klein 1998), and management (Flin 1996; Weick and Sutcliffe 2001).

The sociological contribution focuses mainly on issues of the social construction of risk and the relationship between expert and lay judgements (Beck 1992; Douglas and Wildavsky 1982; Renn 2008; Wynne 1988). Far fewer sociological studies focus on expert decision-making in naturalistic settings. Recent exceptions include Vaughan’s (2021) study of air traffic controllers which examines what makes air traffic control so safe. She tracks the changing relationship between the controllers and the technology they use concluding that the coordination and collaboration skills of the controllers are not replaceable by technology. In contrast, Smith’s (2021) study of engineers in the mining and oil and gas sectors focuses on how engineers think about public accountability in their everyday decision-making. She finds that they view social responsibility as central but must exercise their judgment ultimately through composite corporate forms that sometimes restrict their capacity to act. Our own previous work has shown how workers in hazardous industries adopt narrative-based strategies in their reasoning over the state of the technology and the acceptability of the risk (Hayes and Maslen 2015; Maslen and Hayes 2020).

Following work in the social studies of science that captured how the doing of scientific work involves various “craft” skills (Merz and Knorr-Cetina 1997), even sensory knowledge (Goodwin 1997), there has been a burgeoning interest in the embodied aspects of decision-making in technical work. Daipha’s (2015) account of the decision-making of weather forecasters showed how they are omnivorous when it comes to information; they reach for all pieces of available data provided by remote sensors, but they also just walk outside to observe the weather. Vertesi’s (2015) work on the Mars Exploration Rover showed how the planning and processing of scientific images requires that the scientists and engineers develop ways of seeing with the technology. Vaughan’s (2021) book includes a chapter on embodiment, but she pays surprisingly little attention to the body. Her concern is instead with how being an air traffic controller shapes who they are in their non-working lives.

In this paper we take as our focus the reasoning of pipeline engineers. Engineering as a profession has its origins in the field, with practitioners not only designing structures and machines, but physically building them. Wisnioski (2012, 15) painted us a vivid picture: “Clad in a leather jacket and engineer boots, he (always a he) commanded lesser men, raised dams and factories.” These engineers were craftsmen, who relied on experience, intuition and skill, rather than science (Zhang and Yang 2020). In the more recent history of the profession, engineering has been taken, for the most part, out of the field and into the office. With increasingly complex technologies in the twentieth century, engineering was professionalized and, as part of that, formal study away from the workplace became the norm (Lloyd 2009). By the second half of the twentieth century engineers had become “college-educated residents of suburbia” working “in vast teams with narrow responsibilities” (Wisnioski 2012, 16). Moving from the field into the office brought with it the view that engineering practice is disconnected from the body.

Building on sociological conversations about the relationship between conceptual and embodied thought (Almklov and Hepsø 2011; Leschziner and Brett 2019; Pagis 2010; Vertesi 2015), we examine how these ways of knowing relate in pipeline engineering practice. We have been involved as researchers in the pipeline sector for more than a decade, conducting multiple projects that adopt interview and observational methods. The first author is a sociologist. The second author is also a sociologist but trained first as a chemical engineer working in field and office-based roles for 25 years. This paper draws specifically from an interview-based study that sought to identify key capabilities of Australian pipeline engineers, although our understanding of pipeline engineering practice has built up over a longer period. We show how embodied understanding, acquired through field experience, contributes to engineering reasoning in the office in what is otherwise an archetypally conceptual domain. We take examples from various tasks required of engineers—design, constructability, integrity management—showing how both mind and body come together in these critical areas. Our contribution is to show how embodied reasoning can involve imagining the embodied experience of an other, as opposed to one’s own embodiment. Engineers can see others move, and either imagine themselves doing something similar, or place an imagined self in a future scene.

## Engineering Pipelines

In Australia, and many other countries, buried pipelines cross the country bringing natural gas from the locations where it is extracted from the earth and cleaned in process plants, to densely populated areas where gas is used domestically and in industry. Engineers are involved with pipeline design, construction, operation, and ongoing integrity management to ensure safe and secure provision of energy to domestic and industrial users. While provision of gas is an engineering-intensive activity, engineers often work in small teams that may also include technicians and other professionals such as accountants and policy specialists.

Most engineers work in an office. “The office” refers to sites in which engineers analyze data, do calculations, write procedures, and perform similar tasks linked to design and/or operation of facilities that may be physically far away. To meet the need for safe and reliable supply of energy, they design high-pressure gas pipelines and the associated metering and compression systems and ensure they are constructed and installed correctly. Once the pipelines are operational, engineers ensure that they are monitored appropriately so that any corrosion is detected early and the system can be repaired. Since pipelines are mostly located under streets, suburbs, and farmland, rather than in a controlled industrial area, pipeline engineers also manage the risk of external damage to the pipelines from third parties by providing physical protection measures and by routine or automatic monitoring along the pipeline route.

Much engineering work involves use of computer models to simulate complex systems and assist in the prediction of system behavior. Engineers can invest a great deal of hope and faith in such models, with Trevelyan (2014, 22) going so far as to write that “many engineers yearn for the unerring certainty and feelings of precise control that come from writing their own computer software.” In the pipeline context this can include programs used in design such as software for predicting mechanical stress (and so the propensity for cracking) in piping components subject to internal pressure and cyclic loads. Other computer models assist in operational matters such as interpretation of the results of in line inspection, i.e., measurements made by a sophisticated technical tool run remotely through the pipeline, known as a pig. Engineers use such models to develop an understanding of the likely actual state of the pipeline and so the need to physically dig up sections of the line to inspect it visually and repair if needed.

In addition to these capital city office-based activities, significant work takes place along the pipeline route, known as “the field.” The field can be in a remote rural location where the impact on farming activities or sensitive flora and fauna may be a concern. It can equally be urban streets where interactions with road traffic, railway lines, and other community activities need to be carefully managed. Construction crews use heavy equipment to drill horizontally or excavate trenches for new pipelines. For operational pipelines, personnel patrol the pipeline route by vehicle regularly to look out for anything that might damage the pipeline. This can mean driving on unmade roads in remote locations or literally checking over backyard fences to ensure that easements are maintained. Engineers who work in these locations may be supervising construction, undertaking physical inspections of facilities using specialist tools, or supervising those who regularly patrol pipeline easements.

Although climate change considerations will limit its future use, historically, natural gas has been an excellent fuel for homes and businesses but the very combustion properties that make it a good fuel also mean that it is hazardous if it leaks. Pipeline networks can operate under significant pressure so that a complete rupture of a pipeline is very dangerous. Massive leaks are rare, but the impact of the resultant fire can be catastrophic such as was seen at San Bruno California in 2010 when a pipeline rupture resulted in eight deaths and destroyed 38 homes (Hayes and Hopkins 2014). Pipeline engineering work thus has a particular focus on management of risk, which is addressed as a technical challenge managed via formal decision-making processes (Maslen and Hayes 2020).

With this distinction between the office and the field, the nature of pipeline engineering work has been increasingly viewed as the domain of conceptual reasoning as opposed to embodied know-how. The office-based activities of preparing engineering calculations, specifications, and drawings are seemingly abstract in nature. In contrast, the field is associated with the body as much as the mind. It is dirty, noisy, and exposed to the elements. It is dangerous so special protective clothing is needed. There is an immediacy in this relation between workers and the asset. While there appear to be firm lines between these two domains and the ways of knowing associated with them, there is not such an absolute distinction in practice. However useful models are, they do not directly predict the real world but rather provide inputs into decision-making. While some major equipment is buried and so still hidden from those who work with it, the field can bring engineers up close and personal with these large assets, which are physically in front of them, rather than being lines on a page or words in a document. The realities of the scale of the assets and what it means to work with and around them are key to pipeline engineers’ professional judgments, particularly in relation to risk.

## Knowing with the Mind and the Body

The nature of knowledge and cognition has long dominated the inquiry of philosophers, neuroscientists, anthropologists, psychologists, and sociologists alike. Two main ways of knowing are typically recognized, including conceptual and embodied forms. Following Plato, philosophical contributions in particular have privileged conceptual understanding, with abstract contemplation at the center of the philosophical project (Nussbaum 1986). For Plato, we cannot trust what we know of our world via our senses; it is our rational minds that offer a pathway to truth. A similar line of thought was presented by Descartes, who observed the individual mind as separate from that of the other (that is, a solitary self), and consciousness and reasoning as a matter of “pure intellect,” once more drawing sharp distinctions between one’s mind and body (Descartes 1996). This leads to a view of conceptual knowledge as understanding that can be formalized and stored in its totality (as a formula, as a written idea), and as separate from the knower (Dreyfus 1992).

Greek philosophy also recognized embodied know-how, “practical sense,” or “practical intelligence” (de Certeau 1984). Phenomenological and practice theories draw these aspects of knowing into focus. Merleau-Ponty emphasized embodied exploration as the means through which we know ourselves and our worlds. This exploration can be of one’s own self, as we use one sense or part of our body to explore another, or intersubjective—in seeing how others see we become aware of how we are *doing seeing* (Merleau-Ponty 1962, 1968). Bourdieu’s (1990) influential concept of *habitus* gives account of embodied knowing as habituated and “beyond the grasp of consciousness” (Bourdieu 1977; see also Wacquant 2004). Embodied knowledge has also been lumped in with other forms of tacit knowledge—those aspects of craft or expert understanding that are difficult to articulate or formalize (Collins 1985; Polanyi 1966).

While each has been attended to, conceptual and embodied knowledge forms tend to be treated separately, yet they are intimately intertwined and so we need to attend to how they relate (Cerulo 2015; Ignatow 2007; Lakoff and Johnson 1999; Pagis 2010). Recent contributions have shown creativity is at once analytical and grounded in the body, as in Leschziner and Brett’s (2019) work on how elite chefs develop new dishes. They write that “even the most abstract knowledge—is better understood as grounded in their own flesh and blood” (Leschziner and Brett 2019, 361). Conceptual thinking calls up a sensory experience of the phenomena; it is an exercise in “embodied simulation,” meaning that people imagine the embodied experience while working through something conceptually, as well as anticipating the embodied experience of others who will encounter phenomena later (Gallese and Lakoff 2005; Gibbs 2005). Pagis’s (2010, 471) study of vipassana meditation points to the limits of considering conceptual and embodied ways of knowing separately, showing how Buddhist teachings are highly theorized and the subject of book learning, yet “become lived reality, or truth, only when they become embodied through the practice of meditation.” In both vipassana (Pagis 2019), and the martial art of aikado (Foster 2015), practitioners work through the body to transform the mind. We also see the embodied nature of cognition in our use of language. As Lakoff and Johnson (1999, 36) showed, our use of metaphor shows up how thought relates to our sensory experience, to the degree that our spatial concepts “would not exist if we did not have the kinds of bodies we have.”

Research too on the relation between the human and nonhuman has worked against mind-body dualisms, showing instead the making of bodies and the relationality of knowing. In various domains, we see how the process of becoming sensitive is inseparable from the tools involved in training the body. Goodwin (1994) took the case of archaeologists, showing how their ways of seeing are worked on in relation to the Munsell color chart, which provides an architecture for the perception of dirt. Latour similarly referred to Teil’s work on odor kits involved in training “noses” in the perfume industry. For Latour, the kit itself does much of the work of altering the sense perception of the nose-in-training. He wrote: “All those artificial set-ups are simultaneously layered to make my nose sensitive to differences, namely, to be moved into action by the contrast between two entities” (Latour 2004, 209). Barad (2007) directed attention to the material-discursive arrangements of knowing in different domains. She adopted the term “intra-action” (as opposed to interaction) to focus on relationality and the ontological inseparability of phenomena. Working with these ideas in relation to digital media, Lupton and Maslen (2018) show this intertwining in what they term the “more-than-human sensorium.”

How the conceptual and embodied relate to one another is not universal but varies by epistemic culture (Knorr-Centina 1999). In Micronesian navigation, the local and embodied nature of experience is central, where navigators “estimate the speed of the canoe through direct sensory inputs like the sound of the hull in the water” (Turnbull 2000, 140). And yet, navigation also involves conceptual work, in relation to knowledge of their star compass (*etak*), and seamarks. In scientific work, it is the lab’s “generic placelessness” that allows scientists to study phenomena objectively, and in such a way that experimenters can claim universality to their findings (Kohler 2002, 473). Conducting tests also involves embodied work, with scientists “*repositories of unconscious experience* whose responsibility it is to develop an embodied sense for resolving certain problem situations” (Knorr-Cetina 1992, 119). Against approaches that distinguish between material models and conceptual models (Hacking 1983; Myers 2008, 165, 166) showed how molecular biologists literally get a “*feeling for* proper molecular configuration,” with body-work central to “interpreting the specificities of protein forms and functions.” Both conceptual and embodied knowing may not only be individual, but collective (Vertesi 2012).

We can also observe a temporal dynamic in the relation between conceptual and embodied knowing. When children learn language and other skills, it is practical mastery that comes first, with a conceptual appreciation to follow (if it comes at all) (Greenfield 2000). Training for professional roles begins with university programs in which conceptual knowledge mostly dominates. Medical students can undertake perhaps years of coursework before beginning to develop their clinical skills. Yet, medical and surgical practice is still an apprenticeship, with physicians- and surgeons-in-training undertaking substantial instruction while working with more experienced others following their degree programs. The surgical apprenticeship teaches both the embodied techniques of drilling and cutting, as well as the meaning of the activities (Prentice 2013). This relation can also change in different interactional contexts or phases, as for ballet dancers, where conceptual thinking is more present in practice where dancers analyze how they will be seen and work on their technique. Such conceptual thinking recedes into the background in performance, where the embodied experience of dancing comes to the fore, expressed as “flow” (Kleiner 2009, see also Maslen 2022).

Last, there is a spatial dynamic to knowing, which shows up in the distinction between the field and the office, and the conceptual-embodied work involved in managing their separation. Almklov and Hepsø (2011) showed how geologists working on offshore petroleum exploration and extraction necessarily use analogous field sites that they have been able to visit as a foundation from which to understand the reservoir and so provide the necessary advice. The site that they are working on renders direct human perception impossible, and yet they need an embodied experience to make sense of the geological data that they are presented with. In the office, measured data and models take on a greater significance due to a loss of context. This is a challenge strikingly similar to that faced by the pipeline engineers that we studied. Vertesi showed how the engineers and scientists working on the Mars Exploration Rover Mission perform a “*technomorphic* move” to align their own sense with that of the robot. They move and see as the robot at their desks, placing their hands up to their foreheads “to approximate the location of the Pancam’s eyes” with inanimate objects, like a mobile phone placed on their desk, standing in for “the location of a rock she wants the Rover to image on Mars” ahead of transmitting directions for the following day’s work (Vertesi 2012, 394).

The following analysis extends these lines of inquiry, showing how embodied reasoning can involve embodying the bodies of others and imagined future selves. This relates to the need for engineers to bridge the gap between their own roles and those of construction and operations personnel. It is also a kind of test that engineers can use when reasoning through the social acceptability of risk.

## Methods

### Research Design

The findings in this paper are drawn from a qualitative study of the holistic attributes that help gas pipeline engineers to make good decisions in conditions of uncertainty. We adopt the term capabilities to describe these attributes, as opposed to competence which tends to be more narrowly focused on technical skills (Hayes et al. 2021; Mulder 2014). We investigated these holistic attributes via semi-structured interviews, aiming to identify how pipeline engineers manage the gap between design work in the office and the outcomes of decisions in the field, as well as the skills or approaches that support high-stakes decisions for pipeline engineers, strategies for advocating for outcomes, and ethical dimensions to engineering practice.

We conducted the interviews between August and October 2020. We used videoconferencing for the interviews due to travel restrictions and social distancing requirements during the COVID-19 pandemic. To counter-act some of the potential for a loss of meaning and to manage risk of any failures of the technology (such as a loss of connection by one of the interviewers), all interviews were conducted by two members of the research team. This work was approved by the relevant university human research ethics committees.

### Participants

Recruitment for the interviews targeted practicing and recently retired pipeline engineers in technical roles (rather than managerial or field-based personnel). Most interviewees had spent their professional career working in the downstream gas sector in Australia although some interviewees had international experience. Specific duties included developing asset integrity management plans, running inspection activities and managing repairs, designing new facilities and modifications to existing facilities, supervising construction of new facilities and modifications to existing facilities, undertaking risk assessments, assessing engineering work done by others, and supervising other engineers. Participants’ engineering disciplines spanned mechanical, materials, process, chemical, civil, structural, and electrical and instrumentation, based on their initial qualifications as described to us in interview. All interviewees had undertaken accredited engineering degrees.

Interview participants were recruited in two ways: by direct invitation to pipeline engineers known to the research team due to a decade of previous research with this group; and via email invitation from a mailing list provided by industry partners. Employing organizations are all based in Australia although some of the operating companies are wholly owned subsidiaries of overseas companies and some consulting firms offer their services internationally. This resulted in a total of 41 interviews. No population data is available regarding gender, discipline, or employing organization for the target group as a whole but the interviewee group is broadly representative based on our general knowledge of this group. Details are shown in Table 1.

**Table 1:** Interviewee demographic details

	Number of interviewees	Proportion of interviewees
Gender		
Male	35	85%
Female	6	15%
Discipline		
Mechanical/Materials	26	63%
Process/Chemical	8	20%
Civil/Structural	6	15%
Electrical/Instrumentation	1	2%
Employing organisation		
Operating company	21	51%
Design/construction contractor	14	34%
Regulatory agency	2	5%
Independent consultant	4	10%
Experience		
< 5 years	2	5%
5 - < 10 years	3	7%
10 - < 20 years	17	41%
20 - < 30 years	10	24%
30 years or more	9	22%
Total	41	100%

All interview recordings and transcripts were assigned a project code and cannot be attributed to specific individuals. In the Findings, we identify individual interviewees by their project code.

### Data Analysis

Discussions were audio-recorded and transcribed by a professional transcription company for analysis with the consent of participants. The authors worked together to analyze the transcripts using thematic analysis (Ezzy 2013). In a first pass, the data was coded in NVivo 12 for the engineering capabilities and related themes (see Table 2).

**Table 2:** Engineering capabilities

Capability	Interview themes
Use long term, foresighted reasoning, especially in the face of uncertainty	Imagining worst case outcomes Choosing with the long term in mind Dealing with uncertainty
Understand norms and values that inform actions	Using standards Understanding risk processes and concepts Making ethical choices
Think systematically and understand interconnectedness	Understanding context Prioritizing actions in complex situations Applying risk concepts Taking enough time
Collaborate with and draw on the experience of others	Build networks or seek others’ opinions Knowing your limitations Building a good relationship with field people Building external stakeholder relationships
Ground decisions in reality	Making practical choices Learning from small failures Be skeptical of models and calculations Imagine being there
Advocate for action and take responsibility	Influencing senior management and clients Standing up for public safety

In the course of these discussions, participants talked about their decision-making processes including the value of field experiences. This focus on field experience typically emerged in response to questions about making high-stakes decisions and the temporal and spatial separation between a decision and its outcome. Participants also sometimes described the relationship between office and field-based work in response to the opening question we posed about their professional experience and current role.

For the analysis presented in this paper, we focus on this discussion of field experience as connected to engineering cognition. Typically, such claims are treated as a matter of learning from experience. The positive impact of learning from experience was explained by just under half (n = 17) of the pipeline engineers interviewed, with more describing the role of field experience (n = 26) and learning from cases (n = 27) as part of their professional practice. Amongst these interviewees, there were participants who described learning from their own experiences, participants who described learning from others’ experiences (disaster/accident cases), and those who use their experiences of accidents or those of others to communicate potential risk.

Rather than identifying the degree to which learning from experience is valued, we seek to more closely interrogate these knowledge claims to appreciate why field experience is particularly valuable to engineering decision-making processes, and what this implies for our appreciation of the relationship between conceptual and embodied knowledge in engineering. In particular, we draw together engineers’ accounts of their sensory appreciations of pipelines acquired through field visits, their understanding of grasping the embodied experience of field workers, and the embodied aspects of grasping failure modes.

## Findings

### Sensing the Physicality of the Pipeline

Many pipeline engineers describe the virtues of field experience when reflecting on what makes for robust office-based engineering decisions. In their accounts, sensory appreciations of an asset, specific components, and the surrounding environment feature prominently. This sensory appreciation is part of what is otherwise more abstract thinking back in the office. Going into the field aids in design engineers’ visualization of the asset and what it means to work with it. One engineer put this simply: “First thing is field experience is essential. You need to understand what you’re doing, what it looks like. What looks right, what doesn’t” (I21). One reason that design engineers felt that they benefited from physically seeing the pipeline was to appreciate the scale of design elements. Another engineer explained,

You can see what people are up against out there. It’s kind of hard to visualize unless you’ve kind of gone and had a look. … Like an eight inch valve doesn’t sound very big, right? Because there’s no way you can lift it. You wouldn’t know that unless you went to the field. (I06)

This issue of visualizing the asset relates to the reasoning that needs to happen in the design process. We see the shortcomings of thinking only in conceptual terms in the following account, where an engineer reflects on a design experience years before where he worked on the drawings only, realizing his assumptions were off the mark when later seeing the asset in person. He had not grasped the complexity of his design, the construction challenges that the welder would face, and he too had not appreciated the physical scale of what his drawings would become.

I don’t think there’s any question about the value of field experience. I spent my first year or so in an office and I can still remember you’re looking at drawings and things and then suddenly you go and see them in real life and you think holy shit, I didn’t realize that’s how big it was, or that’s how complex it was, or dealing with subsidy structures how does a welder weld that that I’ve drawn up? That sort of practicability becomes really useful in bringing what’s doable into a design office, there’s no question about that. (I15)

The significance of embodied understanding is evident in cases where it is missing, a common scenario in the case of pipelines buried under the sea floor: “A very distinct challenge of offshore work is we don’t have eyes, we can’t go out, we can’t walk, we can’t poke at it, we can’t dig it up” (I28). In this case, the data points are also few and far between presenting additional challenges, but this engineer said first the lack of ability to go to the field was problematic in and of itself. As we see in this reflection from this engineer, building an embodied understanding of the asset is not only visual in character. It is also about moving around the asset, physically touching it, seeing all the while. Sometimes this embodied understanding is expressed in terms of having a “feel” for the asset in place. As one engineer put it, field experience is important “to actually get a feel for where the pipe is and what’s around it” (I13).

Like Leschziner and Brett’s (2019) elite chefs, the engineers claimed that this embodied understanding endures once they are back in the office. That is, their engineering judgments do not need to take place in the field to result in constructable designs, or work procedures that result in the desired outcome. One engineer explained,

Once there’s understanding as to what things look, see, and feel like, a pipeline engineer can operate from anywhere. It’s getting that initial understanding. Seeing and touching and feeling in the first instance gets that understanding which stays with them for—I’ll say stays with them for life. I look back at some of the things I did 15, 20 years ago, and they’re still clear in my mind, and they probably have an effect on some of the decisions I make today. (I23)

With this in mind, some senior engineers reflected on the strategies that their organizations adopt to support both embodied and conceptual understandings of pipelines. The following engineer described the office and the field as spaces in which conceptual or embodied understanding come in and out of focus, and so the benefits of moving engineers between roles/sites so that they might build these understandings that reside more or less in these spaces.

We flip flop our engineers around a lot, especially when they’re younger, to give them that solid grounding of understanding both the design space and the integrity or the operations space, and I think that’s working in a lot of ways. … The design engineers [based in the office] tend to understand more about the practicalities of their design and how it works in the field, and what problems the operations have, and it’s creating this feedback loop really. And the integrity engineers, or the operation support people [based in the field], they have a stronger understanding of the theoretical basis of what they’re looking at, and why they’re inspecting, and what they’re observing on site, is there a real issue, or the design, did it accommodate what they’re saying? So, for example, just because you’re seeing corrosion doesn’t mean that the corrosion is bad as long as the corrosion rate, and the corrosion allowances, are within the intent of the design. (I28)

Both forms of reasoning are important and so for office-based personnel, their embodied experiences in the field become the lens through which they filter their conceptual work, and vice versa.

### Embodying Others’ Bodies to Grasp Constructability and Operability

**Fig. 1:**
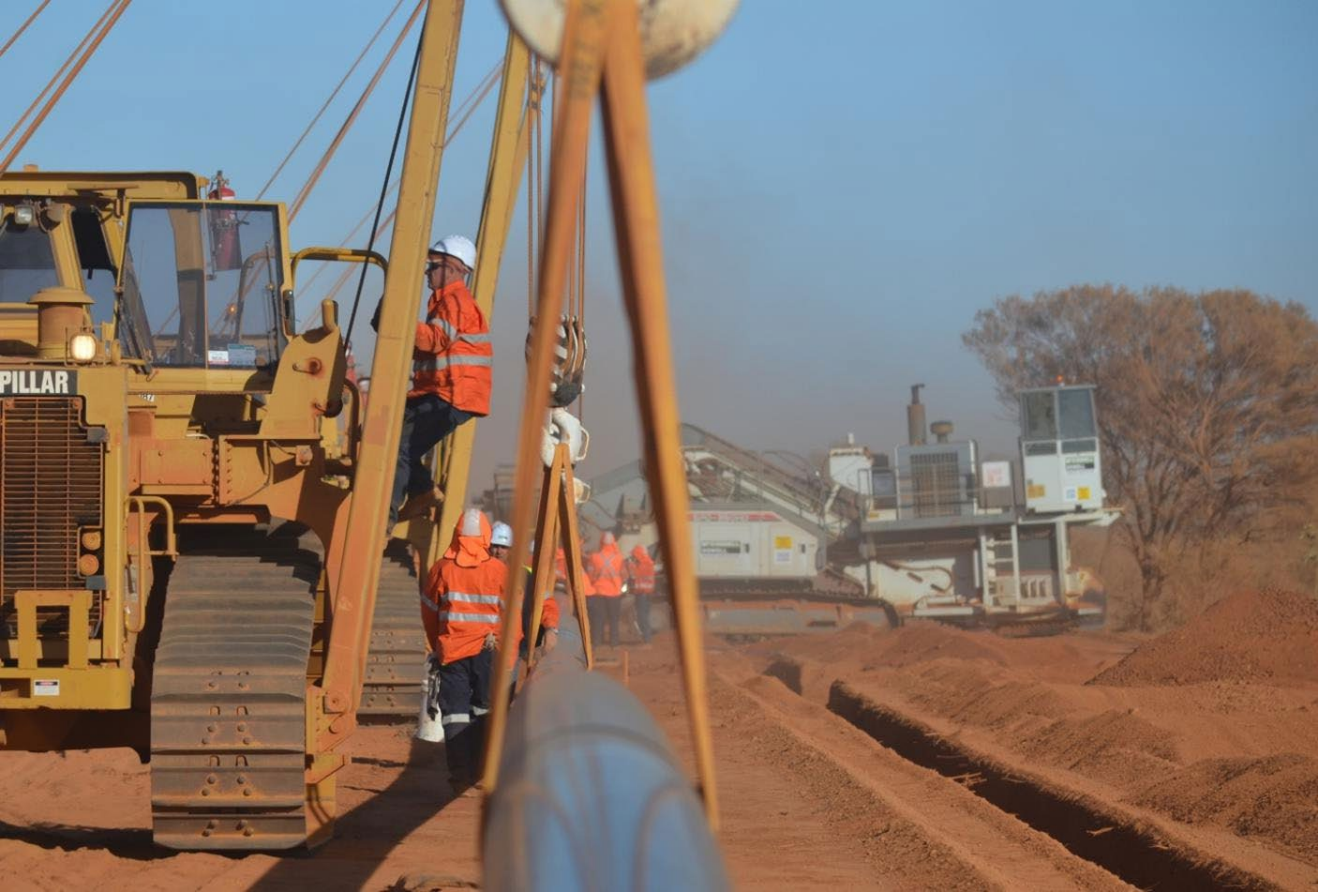
Pipeline construction

Working in the field is not only about building an embodied understanding of the asset and the geography. Embodied understanding extends to an appreciation of the bodies of workers constructing the pipeline. This might seem self-evident, but the people are not on the technical drawings yet they are everywhere once you get into the field (see Fig. 1). Office-based engineers describe moments of realization that there are workers involved in constructing and operating assets. They begin to imagine the area around the asset as including people with agency, with their own embodied affects and potentialities, all of which need to be taken into account.

The example that came up repeatedly is reasoning over whether it is possible for a construction worker to move to perform a weld as drawn. Field experience gives design engineers a sense of how bodies will need to move, if they draw one line on the page, or another.

The best designers would be the ones that have got the construction experience, the ones that have come from the field or not come from the field but spent time in the field. They’re your best designers because they’ve got the practical experience and it’s having that practical experience that says, hang on. … You’ve come up with three welds here but how on earth am I ever going to get in there and do that weld in the middle? It’s physically impossible for me to get an electrode and weld that up, that sort of practical experience when it’s coming to design. But see that’s not a remoteness issue. That’s just experience and practical knowledge. That’s what we need to overcome. (I16)

Note here that in forming this judgment the engineer shifts position from designer to construction worker—“how on earth am I ever going to get in there and do that weld in the middle?” This is a case of taking the role of the other (Mead 1934) to understand whether a design is practically buildable or not.

Especially in reaching an embodied understanding of how others’ bodies will move, they mostly (if not always) appreciate this movement through seeing others, not moving themselves. The following engineer also talks about getting a first-hand appreciation of pipeline construction as an important foundation for design work in the office. In his view, this is not a quick process, achieved through the odd site visit, but emerges through living and working near the construction workers over a longer period: seeing their labor, and the physical challenges that they face.

So if you take the constructability issue, occupational health and safety during the construction phase, you can really only understand that if you’ve seen it firsthand. You’ve kind of got to live it a bit to be able to—you’ve got to go to site for weeks or months and even if you’re not building it yourself witness firsthand what the guys go through to be able to build something, and what is really hazardous or generating risk. And then it’s easy when you’re back in the office in the design phase to go right, I’m not going to design it this way because that will cause this problem on site. (I20)

The value of experience can extend beyond the specific tasks to the work environment too with another engineer who works with offshore pipelines describing how he draws on his experience of challenging work environments. He tries to imagine whether tasks he is designing could be safely performed on a moving ship.

I try to transport myself I guess to some extent, by saying, “Okay, if I can put myself in a situation where I’ve been in similar situations, would I be actually comfortable to actually do that operation?” And I guess that only comes from the fact that I’ve been sitting on construction vessels that have been bouncing around in the waves as we’ve been doing … pigging operations, or doing welding operations on my pipeline. (I15)

Again, here, the engineer is shifting position. As he says, he “transports” himself, inhabiting the body of an imagined operations or construction worker for the purposes of making a judgment about whether they can perform a given task in the given conditions.

The following engineer shared an experience that shows the relationship between workers and the asset and how inadequate consideration of the physical limitations of workers’ bodies resulted in failure of the technology. In this case, there was a change to the procedure for using hand tools to prevent injury to workers. This resulted in new difficulties in one application where the knock-on effects of the change had not been considered.

You’ve got to try and understand the decisions you make at your desk have real-world impact. These things do lead to this, lead to this, lead to this. … you know like the nodding donkey pump, oil pump? I had one of those and because it was a pump and it’s offset, it has a counterweight, and big counterweights to make, you know, so otherwise, you’d need so much power in the pump to lift it up, right? So, one of these counterweights fell off, just kind of fell off and destroyed the fence and hit the wellhead. About three tonnes this counterweight was. So, through the process of doing a tap root [a tool for incident investigation] we kind of worked out that what happened is you generally do them up with a flogging hammer, you know, you put the thing on there, and you hit it, and then people kept breaking their knuckles, so [company] banned flogging hammers. So, then what happened was then it worked out they put it on in such a way that you needed to push upwards on it to get it tight, right? It needed about a thousand newton meters of torque. So, that means that a hundred-kilo guy would have to hang off a one-meter bar to get it tight enough, right? So, if this guy’s up a ladder trying to push upwards, it’s never going to be tight enough. … So, how do you teach that unless you’ve actually seen it, unless you’ve actually followed and seen it happen, seen those steps kind of lead through? (I06)

The engineer who investigated the safety incident caused by the counterweight falling reasoned through the causes by thinking about what the worker would need to have done to ensure it was correctly installed, concluding that the physical action required was impossible if the new procedure was complied with. This same embodied reasoning was not part of the decision-making process for the office-based decision makers who banned the use of flogging hammers, also in the name of safety.

### Embodied Aspects of Grasping Failure Modes

The high-level context to our conversations with engineers was the capabilities necessary to ensure continued public safety. These issues of building, and working from, an embodied sense of the pipeline system were explicitly linked by our interviewees to an appreciation of what can go wrong with pipelines, and so what they need to attend to so as to prevent failures. Most foundationally, field experience draws into sharp focus the very materiality of the systems that office-based engineers design, and their purpose. The following engineer captures such a view, drawing attention to the material that the pipelines transport.

I reckon you need to go to the field … and see what’s going on. Because you kind of, again, someone said to me … “We could be pumping Coca-Cola for all you know,” until you go out and actually realize. (I06)

As described earlier, pipeline failures can be catastrophic causing death and injury to workers and/or members of the public. Most pipeline leaks are small and are repaired without serious consequences but without field experience, office-based engineers can lose sight of both the risks and the consequences. As this engineer put it: “Unless you’ve actually had hands on experience, it’s very difficult to know what the real risks are or the consequences that you might not be immediately aware of.” (I36).

Field experience informs conceptual judgments about the physical state of the system, what can go wrong, and so what they need to do to address it. This engineer describes the danger associated with a short-term view of what makes a good design without considering the longer-term real-world consequences of design office choices.

Maybe it’s just sort of understanding that things fail and they sometimes fail in quite unexpected ways. … When people are doing designs all of the time, they’ll just become very confident in whatever control measures they’re putting in to prevent something happening. And I think what people just don’t think about is how those things degrade over time. And it’s just being aware of “Okay, you’ve put these protections in place, but they can really break down, and you shouldn’t rely on them always being there.” You have to check, maintain them, work out how they might fail and put strategies in place to pick up those failure mechanisms. And I think that should be an important part of design, but it’s often not thought about. You sort of just complete the design and then it gets handed over to the asset owners and they’ve got to work all that stuff out, but it’s not really valued in the project space. (I26)

Another engineer echoed this view of the potential for misunderstanding where engineers rely on computer models alone.

Because I understand and spent a lot of time in the field, I’m able to bridge that potential gap in geography between people making decisions in an office space on what a computer tells them and some idea of how that plays out in the real world. … I’ve seen a lot of fundamentally unsound assessments based on what a computer tells you is possible and that needs an injection of real-world experience. It is definitely a geographically induced problem, but it’s also got to do with the way engineers are gaining their experience at the moment. (I01)

This engineer gave us a specific example of pipeline stress analysis where complex models calculate the potential for failure at joints. Conservative assumptions in the model can give rise to predictions that equipment will fail whereas field experience shows that this is not the case. The value of field experience is thus:

It provides you with a context for understanding what the computer’s telling you, but treating the computer as a calculation tool, not as a thinking device which will tell you whether something’s true or not. And I think if you don’t spend enough time in the real world, where things don’t regularly blow up, and where you see the real limitations of equipment and materials, you’re less likely to fall in for those assumption traps when you sit in front of a computer. You’re more likely to be able to identify what’s probably a false outcome based on what you have seen and experienced. (I01)

The embodied experiences from the field act as a kind of antidote to some of the shortcomings of computer models. The following account in particular points to the multisensory nature of experience that this engineer recalls as he makes high-stakes decisions.

It’s not being rash and prompt in your decision but not procrastinating too much, but also ensuring you’ve got an understanding of the consequences. There’s no point in being an engineer sitting in an office who doesn’t understand the consequence of what they do. To me it’s important for all of our engineers—I’ll probably go a little bit off-track here—to get some field experience so they can understand what they’re looking at, what they’re designing, they can understand the geometry, the magnitude, and that they can get a feeling for the power behind gas, in particular, in transmission pipelines. If something goes wrong, it goes wrong big time mainly. … In terms of those experiences, for me personally it is a simple thing like being in the nearby vicinity—or even seeing and hearing and feeling gas blowdown, and just understanding the power that is there and the energy that is released when you blow down a pipeline. … It’s not textbook. It’s the seeing and feeling. (I23)

The “feel” described here relates not to movement, but to pressure and vibration as felt in the body when there is a release (planned or otherwise) of pressurized natural gas. This appreciation is synesthetic. A gas blowdown is loud, creates pressure waves that can be felt, and it can be seen rushing from the release point too. The gas may be flared, which also creates significant heat that can be felt. The sensate experiences described are linked to what the engineer knows conceptually about how high-pressure gas behaves when released but the embodied experience links this abstract knowledge to the specific danger that must be successfully controlled.

It is also interesting to note here that this engineer felt that in giving this account he was going “off-track.” The answer was not off-track from our perspective as interviewers. The comment perhaps more speaks to the status of embodied ways of knowing in his professional context. It is critical to his reasoning, but in terms of accounting for the reasoning, it is off script.

In some cases, participants had personally experienced accidents, reinforcing in their minds the stakes and what it means for their decision-making processes. Past experiences help engineers to identify patterns that may pose risk. As one expressed: “What feels like gut feeling is in fact experience. That gut feel is generally experience, subconscious experience spotting a pattern, you know, that something might not be quite right” (I06). Professional experience of a major accident was also noted by an interviewee who said, “I was involved in a fatality incident, so you see first-hand what happens and you see the consequences of something failing, and it lasts with you for a long time. … It scared the bejesus out of me, and I started paying attention a lot more after that, I tell you” (I28).

### Imagining a Future Self as a Test of Risk Acceptability

Taking the position of an imagined other is also a tactic in risk assessment. Part of making engineering-based risk decisions involves imagining worst case scenarios, which are almost always about what could happen to someone else—in construction, in operation, or to the public. With this in mind, the engineers are not only thinking about the power of an unplanned release of high-pressure gas abstracted from the people who would experience it if it came to pass. They are also often imagining the physical effects of a disaster on surrounding people for the purposes of reasoning through the acceptability of the risk. As one engineer put it, “Failure comes from failure to imagine failure. If you can’t believe that the passengers on your Thunder River Rapids Ride^1^ might ever be killed, then you’re headed for trouble” (I07).

The imagined other who is exposed to the risk in question often manifests as a future version of self. They imagine this future self physically standing near the facility that they are making a decision about, potentially exposing this self to physical risk, as this engineer expresses:

At the end of the day, you have to live by the decisions you make, and it’s a very good test of, “Would I put myself in that position?” and if you don’t, then it’s not acceptable for someone else at all. (I28)

The following engineer gave an example along these lines, imagining their body beside the operations worker tasked with hot-tapping the pipeline. This is a hazardous activity where a new connection is made to a live system which must be carefully controlled to avoid a major fire.

But then sometimes you think about it afterwards and you go, “Would I be comfortable being there? Would I want to be standing there next to the operations person while they’re doing that?” And that’s kind of—that aspect of it was the only other time that I often think about would I be comfortable standing next to an ops person, and is it because I wouldn’t be comfortable sometimes, is it because of my lack of field experience or is it because I don’t understand the risks properly, so therefore I feel nervous about it, standing there while someone’s hot-tapping the pipeline or something like that. It always makes me feel a bit uncertain even though that whole task is rigidly followed. (I27)

The decision here about whether it is, or is not safe is challenged by this engineer’s lack of field experience. In his embodied imagination it feels scary when he places himself in the scene, but there is a question in his mind over whether this relates to a safety risk to the worker, or a lack of understanding of the risk and its management on his part. The safety literature would suggest he is right to feel uneasy whatever his experience because there is a real risk in doing this type of activity. His embodied imagination is working.

## Discussion

When engineers say that field experience is essential, what they mean is that an embodied appreciation of the asset, the geography, and the people involved is critical to their reasoning processes—this is what they get from the field. Engineers rarely get their hands dirty and yet the physicality of engineering projects looms large. Most of their work involves scoping out activities for others to physically perform. Engineers are often writing, in effect, instructions that get handed on to someone in the field. These are the areas in which we typically acknowledge the importance of field experience. In cases such as this it is not difficult to imagine how field experience would be helpful, giving office-based personnel a clearer grasp of the environment, and the physical nature and challenge of construction and operational work.

The value of field experience is not just to provide the foundation of practically achievable instructions. Design engineers need to see components to appreciate their scale and to understand how these elements function in practice. The need to appreciate what the work site is like, and so how the technology that they are designing will function in the real world given they are large, heavy, dirty, and sometimes noisy objects that must effectively function together and fit in with their surroundings. Engineers lean towards considering the technology abstractly, and yet there is an important lesson here that they are designing infrastructure that will ultimately perform the required function (or not).

These embodied experiences are taken into the office and form part of the reasoning process in what is otherwise an abstract activity. As has been found in other domains, it is not as if an engineer needs to lift an eight inch valve every time they draw one to assess the construction challenge that this might introduce. Rather, reasoning over pipeline construction is an exercise in embodied simulation (Gallese and Lakoff 2005; Gibbs 2005; Leschziner and Brett 2019). In working on a design, they complete calculations, work with standards, work with computer models, and they also recall their embodied memory of what this solution will be like when it is translated from the drawing to an asset surrounded by people in the world.

What is peculiar about the engineering case is their work at taking the role of the other (Mead 1934). There are no people on the technical drawings, or in the models that engineers are often working with. The people (workers, the public) only really exist when engineers interact with them in the field. When working on drawings, or writing procedures, engineers address this shortcoming in the technical artifacts by placing themselves in the hypothetical scene. This gives them a foundation from which to test the robustness of a decision if they, as the engineer, take one course of action, or another.

When working on activities that require them to consider constructability and operability, they are not recalling their own past embodied experience. What they are working on is, as far as possible, embodying the bodies of others. They need to observe how others move on site, they do not perform these operations themselves, and they then take those observations as a ground for this shift in position. We can see this shift from self to other in the language used in the accounts. They do not say, could he, the worker I have seen in the past, move his body like that? They assume the position of the imagined other. Can I fit in that space? How would I manipulate my body to perform a weld in this context? How manageable would I find launching the pig on this moving ship?

Taking the role of the other in this way is part of getting the link between my course of action and theirs. There is a resonance here between what the engineers need to do, and Mead’s (1934) final stage in self-development—the game. The engineers need to internalize all roles and rules, as they relate to the socio-technical system, to grasp what their own conduct needs to be. As Mead (1934, 151) wrote: “He must know what everyone else is going to do in order to carry out his own play,” especially “the roles of those who in some sense control him and on whom he depends” (Mead 1934, 160). It is notable that those engineers who demonstrated the value of field experience to their reasoning were often more senior. In a previous project, the first author met an engineer who had been in the industry for only around a year and was being forced to go into the field. He did not see the point, explaining: “you don’t really learn much by watching a bunch of people dig a hole in the ground.” He was not planning to dig a hole, and he could not, at least at that stage, comprehend how his role relates to the roles of field personnel in that he will need to specify the digging of the hole, and so he is not interested. If we were to continue with Mead’s schema, we might say that this junior engineer is still in the play stage.

They imagine, too, because the outcomes of their decisions are far from certain. One of the critical skills of capable engineers is the use of foresighted reasoning in the face of uncertainty (Hayes et at. 2021). They imagine different courses of action, including how things might go wrong and the potentially catastrophic consequences for workers and the public if they do. In considering catastrophic risks, the engineers often reason via an imagined future version of self. Would I place myself in that position? As we have written about elsewhere, for engineers, risk is first and foremost a technical calculation of probability and consequence (Maslen and Hayes 2020). While the industry is replete with tools to calculate risk, and formal processes to guide the control of risk, it is also the case that uncertainty is inherent in risk assessment, and risk criteria and acceptability are a social construction. Seasoned engineers sometimes try to capture this by saying that risk is a qualitative, not quantitative, judgment. Imagining self as exposed to the risk in question is a test of risk acceptability. It makes the social loom large in what could otherwise end up being an overly technical exercise, with the risk of falling prey to a false confidence through numbers.

This extends our appreciation of the use of an imagined other. Elsewhere, researchers have captured thinking with an imagined other as part of self-presentation, as in the anticipation of how others will interpret positions on land use (Trouille 2014), the management of difficult emotions involved in the work of being a door-to-door salesman (Schweingruber and Berns 2005), or working on musical expression (Maslen 2019). In each of these cases, the individuals who are doing the imagining are working on self in relation to how they will be seen by the other. In the engineering case, it is not so much self-presentation that is at stake. Because of the temporal and geographical separation involved in engineering work, they are making decisions in the present, anticipating a future scenario in which they will likely not be a part. They are more architects of the potential courses of action that will follow, not participants. They assume the role of the other because the engineers are making the decision on their behalf.

These forms of embodied imagination also differ from the cases described elsewhere in the literature. In Vertesi’s (2012) study, embodied imagination is “technomorphic” in character, as the engineers and scientists working with the Mars Rover take their own bodies as a proxy for the machine. Our engineers are not imagining they are a pipeline. In a sense, what they perform is the opposite of a technomorphic move. They are humanizing the socio-technical system as it manifests in the technical artifacts that they work with.

Lastly, we need to account for the precise relationship between embodied and conceptual reasoning in the case of engineering. However important, in a variety of ways, embodied knowing is subservient to the conceptual. In terms of sequence, engineers are typically building conceptual knowledge first in university programs. They are not coming out of the field, as do bush firefighters (Neale and May 2020). They then may have an opportunity to visit the field, with this embodied way of knowing giving context and perhaps a reality check to their conceptual understanding.

In terms of a decision-making process too, conceptual knowledge is primary. Engineers typically start with calculations, models, and drawings, later moving between their embodied and conceptual understanding to test whether what they have arrived at abstractly is right. In terms of articulating a judgment in writing or in conversation with peers, what involves an embodied way of knowing needs to be developed and translated into a technical artifact. Especially with respect to risk controls, the engineers say they avoid referring to a potential physical threat to others in terms of death and destruction when making the case for expenditure on a design feature or remedial action (Maslen et al. 2021). Imagining the disaster, the physical experience of it, is internal. The embodied imagination of the worst case as it could happen to others is then the ground for another conceptual reasoning process about likelihood, and what would need to be done in a technical sense to address it. The reasoning is then only articulated technically. This is perhaps why in over a decade of observing and interviewing these engineers we have collected little data on the embodied aspects of engineering. Like Daipha’s (2015) weather forecasters, the engineers are data omnivores, but this is almost a closet practice.

These new insights into embodied aspects of engineering decision-making have an important practical contribution. Despite the need for the embodied appreciation of an asset and the workers, opportunities to go to the field are not available in all workplaces, and so office-based engineers can go about their role without that embodied understanding. The nature of the profession is changing, with even greater distinctions between the office and the field. It was noted by some that field experience has become undervalued over time by industry given the apparent lack of immediate benefit or purpose. In this paper we have demonstrated the value of embodied understanding in pipeline engineers’ reasoning, presenting evidence to support the claim that time in the field should not be considered as an optional add-on if we want to support continued robust technical decisions in the face of uncertainty.

## Footnotes

1 The interviewee is referring to a fatal accident at Australia’s largest theme park, Dreamworld. In 2016, a raft carrying six people in the white-water rafting ride *Thunder River Rapids* collided with an empty raft causing it to flip. Four adults died. The Coroner’s Report found that the design and construction of the ride, along with “multiple significant modifications” between 1987 and 2016 that were made without consideration of hazard consequences, posed significant safety risks for patrons.

## References

[CR1] Almklov Petter G, Vidar, Hepsø (2011). Between and beyond data: How analogue field experience informs the interpretation of remote data sources in petroleum reservoir geology. Social Studies of Science.

[CR2] Barad Karen (2007). Meeting the universe halfway: Quantum physics and the entanglement of matter and meaning.

[CR3] Beck Ulrick (1992). Risk society: Towards a new modernity.

[CR4] Bourdieu Pierre (1977). Outline of a theory of practice.

[CR5] Bourdieu Pierre (1990). The logic of practice.

[CR6] Cerulo Karen A (2015). The embodied mind: Building on Wacquant’s carnal sociology. Qualitative Sociology.

[CR7] Collins Harry (1985). Changing order: Replication and induction in scientific practice.

[CR8] Daipha Phaedra (2015). Masters of uncertainty: Weather forecasters and the quest for ground truth.

[CR9] de Certeau Michel (1984). The practice of everyday life.

[CR10] Descartes Rene (1996). Meditations on first philosophy: With selections from the objections and replies.

[CR11] Douglas Mary (1982). Risk and culture: An essay on the selection of technological and environmental dangers.

[CR12] Dreyfus Hubert (1992). What computers still can’t do: A critique of artificial reason.

[CR13] Flin Rhona (1996). Sitting in the hot seat: Leaders and teams for critical incident management.

[CR70] Ezzy, Douglas. 2013. *Qualitative analysis*. London: Routledge.

[CR14] Foster Drew (2015). Fighters who don’t fight: The case of aikido and somatic metaphorism. Qualitative Sociology.

[CR15] Gallese Vittorio (2005). The brain’s concepts: The role of the sensory-motor system in conceptual knowledge. Cognitive Neuropsychology.

[CR16] Gibbs Raymond (2005). Embodiment and cognitive science.

[CR17] Goodwin Charles (1994). Professional vision. American Anthropologist.

[CR18] Goodwin, Charles. 1997. The blackness of black: Color categories as situated practice. In *Discourse, tools and reasoning: Essays on situated cognition*, eds. Lauren B. Resnick, Clotilde Pontecorvo, and Roger Säljö, 111–140. Berlin: Springer Berlin Heidelberg.

[CR19] Greenfield, Patricia. 2000. Children, material culture and weaving: Historical change and developmental change. In *Children and material culture*, ed. Joanna Sofaer Derevenski, 72–86. London; New York: Routledge.

[CR20] Hacking Ian (1983). Representing and intervening: Introductory topics in the philosophy of natural science.

[CR21] Hayes Jan (2014). Nightmare pipeline failures: Fantasy planning, black swans and integrity management.

[CR22] Hayes Jan, Maslen Sarah (2015). Knowing stories that matter: Learning for effective safety decision-making. Journal of Risk Research.

[CR23] Hayes Jan, Maslen Sarah, Holdsworth Sarah, Sandri Orana (2021). Defining the capable engineer: Non-technical skills that support safe decisions in uncertain, dynamic situations. Safety Science.

[CR24] IAEA Director General (2015). The Fukushima Daiichi accident.

[CR25] Ignatow Gabriel (2007). Theories of embodied knowledge: New directions for cultural and cognitive cociology?. Journal for the Theory of Social Behaviour.

[CR26] Janis Irving (1982). Groupthink: Psychological studies of policy decisions and fiascoes.

[CR27] Klein Gary (1998). Sources of power: How people make decisions.

[CR28] Kleiner Sibyl (2009). Thinking with the mind, syncing with the body: Ballet as symbolic and nonsymbolic interaction. Symbolic Interaction.

[CR29] Knorr-Centina Karin (1999). Epistemic cultures: How the sciences make knowledge.

[CR30] Knorr-Cetina Karin, Pickering Andrew (1992). The couch, the cathedral and the lab: On the relationship between experiment and laboratory science. Science as practice and culture.

[CR31] Kohler Robert (2002). Labscapes: Naturalizing the lab. History of Science.

[CR32] Lakoff George, Johnson Mark (1999). Philosophy in the flesh: The embodied mind and its challenge to Western thought.

[CR33] Latour Bruno (2004). How to talk about the body? The normative dimension of science studies. Body & Society.

[CR34] Leschziner Vanina (2019). Beyond two minds: Cognitive, embodied, and evaluative processes in creativity. Social Psychology Quarterly.

[CR35] Lloyd, Brian. 2009. A short history of professional engineers in Australia. Ingenieurs Australia Society. Accessed on 11 April 2021. https://www.profengaust.org.au/articles/short-history-professional-engineers-australia/#:~:text=The%20number%20of%20persons%20recognised,mainly%20serving%20the%20mining%20industry.

[CR36] Lupton Deborah, Maslen Sarah (2018). The more-than-human sensorium: Sensory engagements with digital self-tracking technologies. The Senses & Society.

[CR37] Majority Staff of the Committee on Transportation and Infrastructure (2020). Final committee report: The design, development and certification of the Boeing 737 Max.

[CR38] Maslen Sarah (2019). Playing with imagined others: Developing a musical ear in conversation with recordings. Symbolic Interaction.

[CR39] Maslen Sarah (2022). Hearing like a musician: Integrating sensory perception of self into a social theory of self-reflexivity. Social Psychology Quarterly.

[CR40] Maslen Sarah (2020). This is how we debate”: Engineers’ use of stories to reason through disaster causation. Qualitative Sociology.

[CR41] Maslen Sarah, Hayes Jan, Holdsworth Sarah, Sandri Orana (2021). When ethics is a technical matter: Engineers’ strategic appeal to ethical considerations in advocating for system integrity. Science and Engineering Ethics.

[CR68] Merleau-Ponty, Maurice. 1962.* Phenomenology of perception*. London; New York: Routledge.

[CR69] Merleau-Ponty, Maurice. 1968.* The visible and the invisible*. Evanston: Northwestern University Press.

[CR42] Mead Geroge Herbert (1934). Mind, self, and society: From the standpoint of a social behaviorist.

[CR43] Merz, Martina, and Karin Knorr-Cetina. 1997. Deconstruction in a ‘thinking’ science: Theoretical physicists at work. *Social Studies of Science* 27 (1): 73–111.

[CR44] Mulder Martin, Billet Stephen, Harteis Christian, Gruber Hans (2014). Conceptions of professional competence. International Handbook of Research in Professional and Practice-based Learning.

[CR45] Myers Natasha (2008). Molecular embodiments and the body-work of modeling in protein crystallography. Social Studies of Science.

[CR46] National Commission on the BP Deepwater Horizon Oil Spill and Offshore Drilling. 2011. *Deep water: The Gulf oil disaster and the future of offshore drilling*. National Commission on the BP Deepwater Horizon Oil Spill and Offshore Drilling. https://www.govinfo.gov/app/details/GPO-OILCOMMISSION. Accessed on 11 October 2017.

[CR47] Neale, Timothy, and Daniel May. 2020. Fuzzy boundaries: Simulation and expertise in bushfire prediction. *Social Studies of Science* 50 (60): 837–859. 10.1177/0306312720906869 32053028

[CR48] Nussbaum Martha (1986). The fragility of goodness: Luck and ethics in Greek tragedy and philosophy.

[CR49] Pagis Michal (2010). From abstract concepts to experiential knowledge: Embodying enlightenment in a meditation center. Qualitative Sociology.

[CR50] Pagis Michal (2019). Inward: Vipassana meditation and the embodiment of the self.

[CR51] Polanyi Michael (1966). The tacit dimension.

[CR52] Prentice Rachel (2013). Bodies in formation: An ethnography of anatomy and surgery education.

[CR53] Renn Ortwin (2008). Risk governance: Coping with uncertainty in a complex world.

[CR54] Schweingruber David (2005). Shaping the selves of young salespeople through emotion management. Journal of Contemporary Ethnography.

[CR55] Smith Jessica (2021). Extracting accountability: Engineering and corporate social responsibility.

[CR56] Suhr Jim (1999). The choosing by advantages decisionmaking system.

[CR57] Trevelyan James (2014). The making of an expert engineer.

[CR58] Trouille David (2014). Fencing a field: Imagined others in the unfolding of a neighborhood park conflict. City & Community.

[CR59] Turnbull David (2000). Masons, tricksters and cartographers: Comparative studies in the sociology of scientific and indigenous knowledges.

[CR60] Vaughan Diane (2021). Dead reckoning: Air traffic control, system effects, and risk.

[CR61] Vertesi Janet (2012). Seeing like a rover: Visualization, embodiment, and interaction on the Mars Exploration Rover Mission. Social Studies of Science.

[CR62] Vertesi Janet (2015). Seeing like a rover: How robots, teams, and images craft knowledge of Mars.

[CR63] Wacquant Loïc (2004). Body and soul: Notebooks of an apprentice boxer.

[CR64] Weick, Karl, and Kathleen Sutcliffe. 2001. *Managing the unexpected: Assuring high performance in an age of complexity*. San Francisco: Jossey-Bass.

[CR65] Wisnioski Matthew (2012). Engineers for change: Competing visions of technology in 1960s America.

[CR66] Wynne Brian (1988). Unruly technology: Practical rules, impractical discourses and public understanding. Social Studies of Science.

[CR67] Zhang, Ce, and Jianming Yang. 2020. *A history of mechanical engineering*. Singapore: Springer.

